# Is arthrocentesis of temporomandibular joint with corticosteroids beneficial? A systematic review

**DOI:** 10.4317/medoral.21925

**Published:** 2018-04-24

**Authors:** Amin Davoudi, Hossein Khaki, Iman Mohammadi, Mehran Daneshmand, Alireza Tamizifar, Meysam Bigdelou, Farzin Ansaripoor

**Affiliations:** 1 Post graduate student of Prosthodontics, Department of Prosthodontics, Dental School, Isfahan University of Medical Sciences, Isfahan, Iran; 2 Post graduate student of Orthodontics, Department of Orthodontics, Dental School, Mashhad University of Medical Sciences, Mashhad, Iran; 3 Assistant professor of Oral and Maxillofacial Surgery, Department of Oral and Maxillofacial Surgery, Dental School, Isfahan University of Medical Sciences, Isfahan, Iran; 4 Post graduate student of Oral and Maxillofacial Surgery, Department of Oral and Maxillofacial Surgery, Dental School, Isfahan University of Medical Sciences, Isfahan, Iran; 5 Post graduate student of Oral and Maxillofacial Surgery, Department of Oral and Maxillofacial Surgery, Dental School, Mashhad University of Medical Sciences, Mashhad, Iran

## Abstract

**Background:**

Temporomandibular disorders (TMDs) are musculoskeletal conditions that can inhibit the normal function of temporomandibular joints (TMJs) and affect the patient’s quality of life, negatively. Arthrocentesis (AC) is a minimally invasive surgical procedure used for treating TMDs. The aim of present paper is to evaluate the advantages of administrating corticosteroid (CS) during AC by reviewing high quality released articles.

**Material and Methods:**

Searching on Cochrane Library, Web of Science, Google Scholar, PubMed, ProQuest, and Scopus databases were performed with focusing on proper key words. Related titles and abstracts, up to December 2017, were screened and selected based on inclusion criteria. The full text of all randomized controlled trials (RCTs) was extensively read and subjected to quality assessments.

**Results:**

After initial search, a total of 2067 articles were included into the study. Finally, 7 studies were reliable enough in methodology and randomization to be included into the study. All of the observed studies showed improvements in jaw functions and pain relief with no statistical differences in both AC and control groups. One study reported painless maximum incisal opening in CS group than the control group.

**Conclusions:**

Based on available RCTs, the AC of TMJ with CS seems to result in similar findings to other therapeutic drugs, with no significant differences.

** Key words:**Arthrocentesis, corticosteroid, temporomandibular joints, temporomandibular joint disorders.

## Introduction

Temporomandibular disorders (TMDs) are multifactorial musculoskeletal conditions that alter the normal function of temporomandibular joints (TMJs), masticatory musculature, and surrounding soft tissues that might result in severe pain and limitation of normal jaw motions (1). Based on reports, about 5-12% of the general population suffer from the TMDs which is also mostly common in women (2). The consequences of TMDs can affect the patient’s quality of life (QoL) as TMJs are often solicited during daily activities such as chewing and speaking (3). The etiologies of TMDs are not consensus. Developmental situations, trauma, infection, immunologic conditions, and neoplasms are some of those suggested etiologies. Nevertheless, bruxism, clenching, stress, and malocclusion are the most common reasons of TMDs referred to the clinicians (4).

Several treatment strategies are introduced up to date. These treatments are also varied from conservative therapies to surgical procedures. Conservative therapies are, for instance, occlusal splints therapy, myofunction therapy, at home exercises, manual therapies, biofeedback and acupuncture (5). The exact mechanism of action of these therapies are not clarified yet. Some authors suggested combination of several peripheral, central and behavioral positive effects are as the result of these therapeutic methods (6). Decreased anxiety, depression, pain intensity, muscle sensitivity, and cognitive awareness are some possible effects of these treatments (5). Arthrocentesis (AC) is another conservative treatment, like previous mentioned therapies, which can be ordered as both first or second line of treatment plan. During splint therapy, for instance, long term treatment period is ordered which might delay the achievement of efficient therapy and results in persistent soft tissue arthropathy. In this situation, the AC may have superior indication as first line treatment plan (7). Also, if other treatments failed, AC may be administered as second line of treatment plan (8). AC is a minimally invasive surgical treatment used for treating TMDs specially in patients with internal derangement of the TMJ. The AC procedure mostly relies on intra-articular washing with some therapeutic drugs such as normal saline, corticosteroids (CS), Hyaluronic acid (HA) and etc. (9). Through AC procedure, the following important substances might be washed away from the joint space: the microscopic necrotic tissue debris, inflammatory by-products, degraded tissues, and inflammatory enzymes and mediators (10). One study investigated the levels of interleukins (1β, 6, 8, and 11), and tumor necrosis factor-alpha in synovial fluid before and 2 weeks after AC (11). They stated that all of mentioned mediators were significantly decreased after 2 weeks of observation. AC also stimulates the normal synovial secretion by lubricating and eliminating the inflammatory mediators which helps the TMJ to turn into its normal range of motion (10,11). Even by returning the TMJ to its normal function, the regeneration and reconstruction process of articular tissues would be initiated after AC (10,11).

As mentioned above, different therapeutic drugs are suggested to be associated with AC. A recent systematic review, conducted by Goiato *et al.*, evaluated the effects of using HA for AC (12). After final evaluation, they concluded that AC with HA is beneficial in improving the pain and functional symptoms of TMDs. They recommended clinicians to evaluate the use of CS and non-steroidal anti-inflammatory drug to find more precise results in future studies (12).

CSs are anti-inflammatory drugs that interrupt the inflammatory and immune pathways. They have been used for both therapeutic and diagnostic purposes. Also, they showed their alleviating effects by suppressing inflammatory responses (13).

The intra-articular use of CS has been tested by some studies (14,15). Samiee *et al.* evaluated the use of intra-articular injection of CS and local anesthesia in patients with disc displacement without reduction (14). Their final results were hopeful and positive responses were observed. Similar results were found by Giraddi *et al.* who evaluated the use of AC with CS in patents with internal derangements of TMJ (16). They proceeded AC with using Ringer lactate followed by injection of either betamethasone or sodium hyaluronate into the TMJ space. They stated that both betamethasone and sodium hyaluronate showed similar results after AC (16). In contrast, Olsen-Bergem and Bjørnland reported that the use of AC with CS is not useful in patents with TMD (17). They tried triamcinolone hexacetonide as the test CS in patients with juvenile idiopathic arthritis. They reported that adding a CS to AC procedure is not beneficial enough (17).

As the data about the use of AC with CS seem to be sparse and there is no systematic review on this scope available, the aim of present review study is to answer the following PICO question (P: participant; I: intervention; C: comparator; O: outcome): What are the effects of AC with CS in patients with any kinds of TMDs, compared to other methods of TMD therapy, in improvements of signs and symptoms? Also, the null hypothesis of this study was to assess whether administration of AC with CS leads to significant improvements of TMD symptoms rather than other therapeutic methods or not.

## Material and Methods

-Study design:

To enhance structural reporting of the articles, the reviewing setting was in accordance to the Preferred Reporting Items for Systematic Reviews and Meta-Analyses (PRISMA) guidelines (18).

Firstly, a clinical question was defined for screening the qualified clinical studies based on PICO: Patients with any kinds of TMDs (P, population) who underwent AC with CS (I, intervention), compared to other methods of TMD therapy (C, comparison) that causes the improvement of signs and symptoms (O, outcome).

A data search was performed using Cochrane Library, Web of Science, Google Scholar, PubMed, ProQuest, and Scopus databases of articles, based on the defined MeSH and non-MeSH terms in simple or multiple conjunctions ( Table 1). The searching procedure was conducted manually up to December 2017, then Endnote software version 7 (Thomson Reuters, NY, USA) was used for final confirmation, cross matching, and avoiding any missing of data.

**Table 1:**
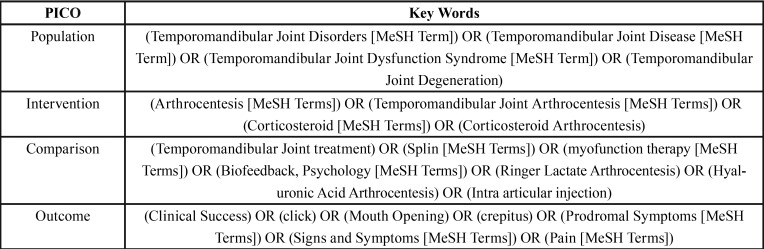
Applied PICO keywords.

Two independent reviewers (A.D and F.A) qualified the eligible articles to review. To select the studies, all obtained English language reports were reviewed, and titles and abstracts were screened for relevance. The review articles and references from different studies were used to identify relevant articles. In the case of disagreement between reviewers, a discussion was undertaken until mutual agreement was reached. Reviewers’ agreement was tested with the Cohen κ test by use of MedCalc software (MedCalc Software, Ostend, Belgium) (kappa score = 0.89).

The studies were subjected to Jadad Score Calculation for Critical Appraisal and lowering the risk of biases (19). They were classified as follow: 1-2 low quality, 3 moderate, and 4-5 high quality ( Table 2). The full text of relevant abstracts was obtained and selected using the following inclusion and exclusion criteria.

**Table 2:**
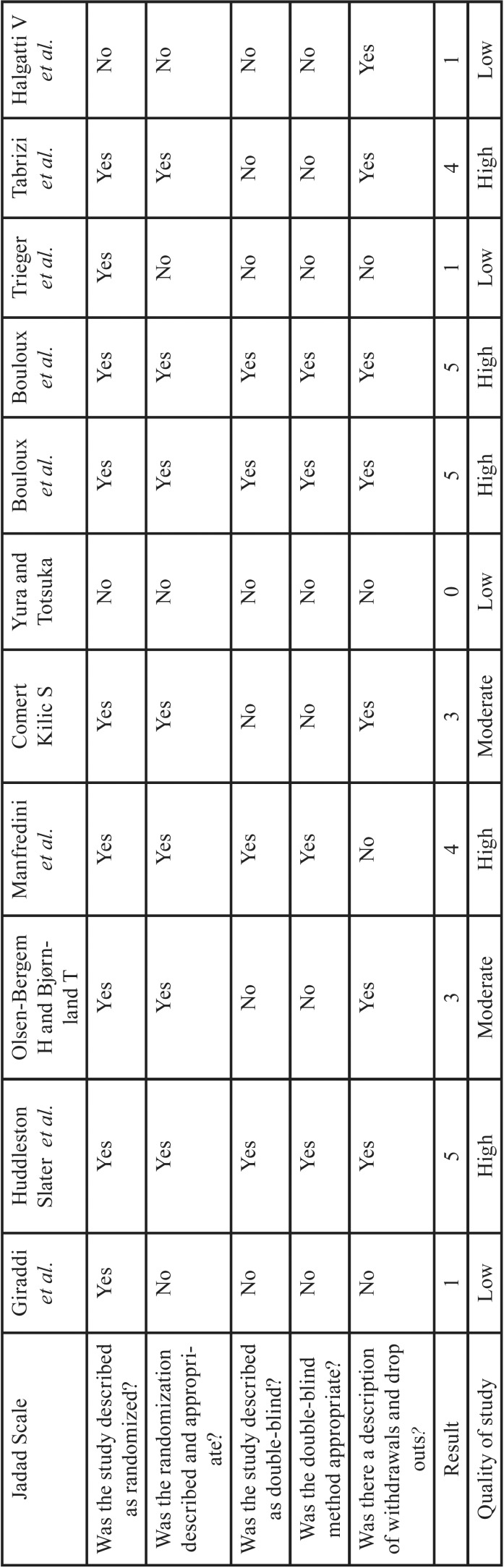
Jadad Score Calculation of selected studies.

-Inclusion criteria.

• English language randomized clinical trials (RCTs) and prospective studies that investigated the effect of TMJ AC with CS

• Clinical research on at least 5 patients

• Maintaining the standard indications and guidelines of AC procedure 

• Performed at least one standard test for evaluating clinical effects or side effects of CS 

-Exclusion criteria.

• Case reports

• Animal studies

• Studies with missing data

• Repeatedly published studies; the last version was included

• Studies in languages other than English

• Studies with Jadad score of < 3 (for eliminating the risk of biases)

**Figure 1:**
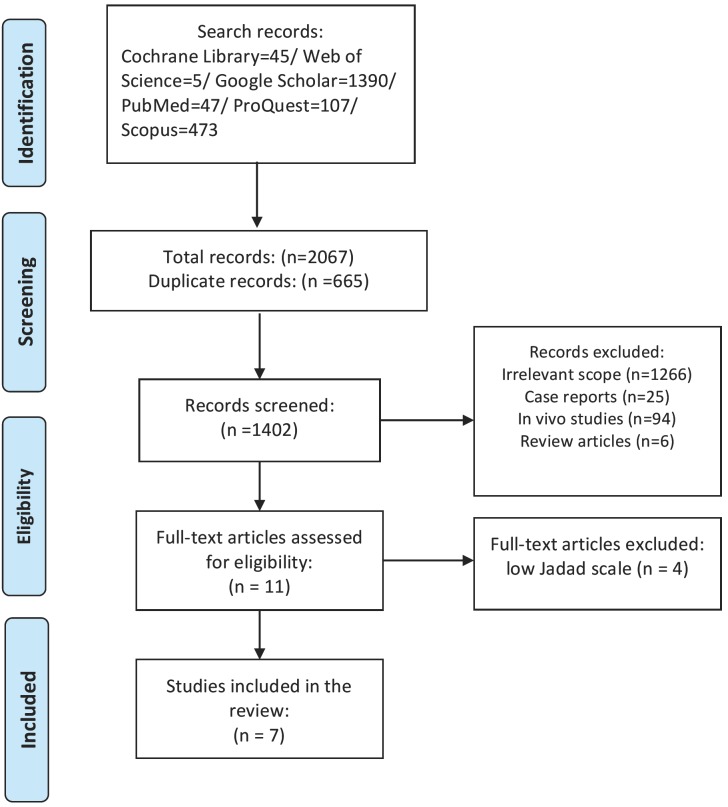
The flowchart of searching strategy based on PRISMA guidelines.

The initial literature search yielded on 2067 articles (Cochrane Library=45/ Web of Science=5/ Google Scholar=1390/ PubMed=47/ ProQuest=107/ Scopus=473), in which 1402 articles remained after removing duplicates. After the first screening based on the title and abstract, 11 studies (13,16,17,20-27) were found eligible which reached to 7 studies (13,17,20-24) after excluding high risk articles (Fig. 1). Full-texts of the all articles were reachable for initiating reviewing process.

-Data extraction:

The following data were collected for each study: author, year, study design, participants (age, gender), method of TMD diagnosis, administered CS and dosage, the monitoring tests before and after AC, clinical significant outcomes. After gathering information, the possibility of preparing a meta-analysis was judged by an independent statistician and epidemiologist. As the collected data were vastly heterogeneous (like different corticosteroid drugs with different dosages, different diagnosis of TMD, different clinical test on the patients, and etc.) no meta-analysis were prepared.

## Results

A total of 2067 articles were included into the study after initial search. 1402 articles were remained after removing the duplicated ones of which 11 studies were eligible to be screened. The full texts of these articles were gathered and each one fulfilled the inclusion criteria were observed. Relying on the Jadad scale ( Table 2), three studies had a score of 5 (20-22), two had a score of 4 (23,24), and two had a score of 3 (13,17), for a total of 7 high quality studies. The rests, which had low Jadad scale, were excluded from the study to avoid biases.

All of the reviewed studies were RCTs (13,20-24), in which two of them were single blinded (13,24), one cohort study (17), and the rests were double blinded (20-23). The total number of patients enrolled in the studies was 397, ranging from 11 to 51 years old ( Table 3).

**Table 3:**
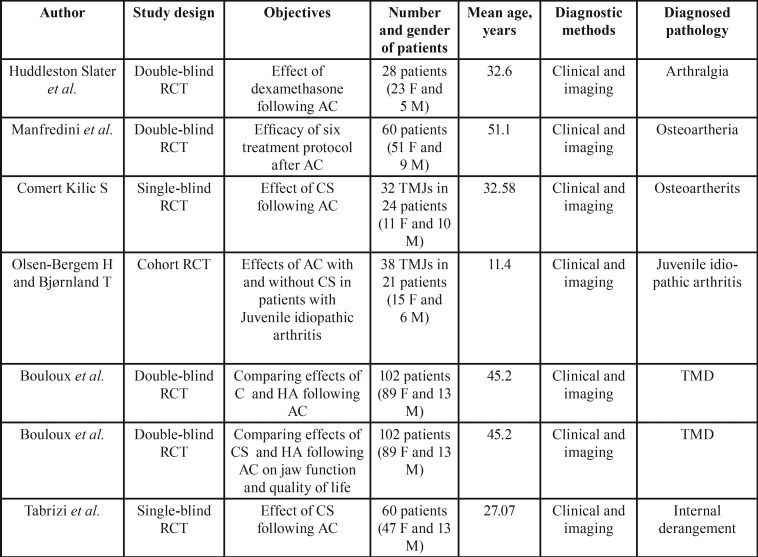
Details of reviewed article.

Regarding the clinical problem, one study evaluated patients with arthralgia (22), two studies focused on osteoarthritis (13,23), one on internal derangement (24), one on Juvenile idiopathic arthritis (17), and the others observed TMDs with no specific disorder (20,21) ( Table 3). Among reviewed studies only 4 studies used the Wilkes classification criteria (13,20,21,23). All of these researches included patients with axis 1 group III for AC intervention.

Regarding to the therapeutic methods and types of treatment ( Table 4), three studies compared the use of HA with CS in their study groups (20,21,23), the others only investigated the effect of AC with CS compared to ringer lactate (13,20,21), or normal saline (22,23), or Vitamin B12 + physiological salt water (17), as a control medium. The follow-up period among the studies ranged from 1 month to 8 months.

**Table 4:**
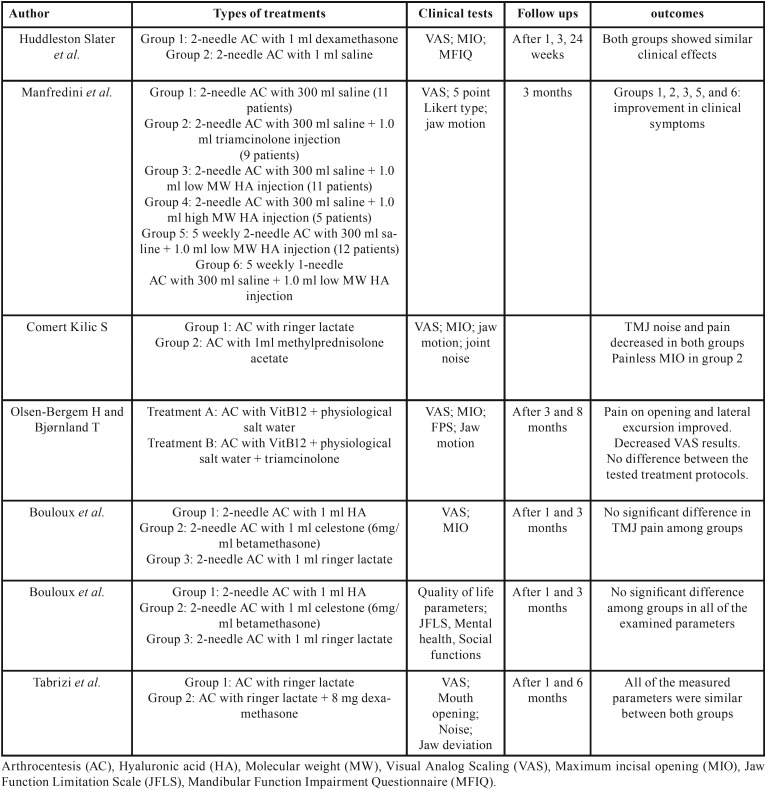
Study grouping, clinical tests, and clinical significant results of reviewed articles.

Regarding to the studies’ outcomes, no consensus result was found about the paramount effect of using CS in AC for the treatment of TMDs (20,21). In a study by Comert Kilic S (13), the CS group, which received methylprednisolone acetate along AC, exhibited better results in terms of painless maximum incisal opening (MIO) than the control group. In another study conducted by Manfredini *et al.* (23), all of the observed groups showed improvements in jaw functions and pain relief with no statistical differences, however the group 4, whom received AC with high molecular weight HA injection, showed negative results (23).

Although seven RCTs were included in this review, no systematic review or meta-analysis was performed about the efficacy of AC with CS with definite results.

## Discussion

Recent systematic reviews on the effect of AC with HA (12), and intra-articular injection (not AC) of CS (28) have seemed effective for treatment of TMDs. However, no systematic review has been dedicated to the use of CS for AC. The null hypothesis of this study was to review whether administration of AC with CS leads to significant improvements of TMD symptoms rather than other therapeutic methods or not. Since there were no consensus findings among the screened studies, the null hypothesis was rejected. Nevertheless, the results do not deny the effectiveness of AC with CS in treatments of TMDs and seems to have similar effectiveness like other methods.

Based on Jadad scale, only 7 studies (out of 11) were reliable enough in methodology and randomization to be included in the study for avoiding any risk of biases.

Wilkes classified the TMDs and internal derangements of TMJ according to the clinical and radiological progressions as follow: early (I), early intermediate (II), intermediate (III), late intermediate (IV), and late (V) stages (29). Also, there is Research Diagnostic Criteria for Temporomandibular Disorders (RDC/TMD) validation project for comprehensive assessment of TMD classification (30). All of the four studies, which used Wilkes criteria, enrolled patients with Axis 1 group III. The Axis 1 is categorized into 3 subgroups: group I: muscle disorders; II: disk displacements; III: Arthralgia, Arthritis, Arthrosis (30).

As mention before, AC eliminates the inflammatory by-products of synovial fluids and stimulates the normal lubrication properties of synovial membrane (31).

The anti-inflammatory role of CS is through interrupting immunological pathways which has been proved in medical texts (32,33). CS therapy affects the synovial tissues by suppressing the inflammatory molecules and passing through the cellular membrane and binding to CS receptors located in the cytoplasm. These activated receptors eliminate the expression of pro inflammatory cytokines, enzymes, and inflammatory process (13). One of the most important anti-inflammatory mechanism seems to be related to the upregulation and production of lipocortin 1. In consequence, the phospholipase A2, cyclooxygenase 1 and 2, and lipooxygenase are blocked which leads to decrease in the production of prostaglandins, prostacyclins, and leukotrienes (20).

Recent tissue culture researches on the effects of CSs revealed that they are capable to depress the degradation of cartilage harvested from patients with osteoarthritis and rheumatoid arthritis. The inhibition of various inflammatory mediators and decrease in the activity of proteolytic enzymes might be the main reason (13).

Methylprednisolone acetate, triamcinolone hexacetonide, triamcinolone acetonide, betamethasone acetate, betamethasone sodium phosphate, betamethasone dipropionate, betamethasone sodium phosphate are the most administered intra-articular CSs (34). Amongst them, betamethasone is more administered for intra articular injection in TMDs, however methylprednisolone acetate, which is more prescribed in the United States, is mostly associated with triamcinolone hexacetonide and triamcinolone acetonide for intra articular injections (35). Among the reviewed studies, betamethasone was used in two studies (20,21), and methylprednisolone acetate was injected in one study (13). The others used dexamethasone (22,24) and triamcinolone (17,23). Some studies declared that methylprednisolone acetate represents better healing potential on articular surfaces. It also is more soluble with prolonged effectiveness (36). However, in a recent in vivo study, the cytotoxicity of betamethasone acetate, methylprednisolone acetate, triamcinolone acetonide were compared and the results manifested that betamethasone acetate and methylpresdnisolone acetate were chondrotoxic and synoviotoxic in contrast to triamcinolone which caused more viable effects (37).

Huddleston *et al.* tried dexamethasone for AC with this rationale that it modifies the vascular regeneration by eliminating both destructive enzymes and the actions of inflammatory cells (22). However, their results did not show either positive or negative effects of dexamethasone. They believed that uncertainty in the working time of dexamethasone, which is about 36-72 hours, makes the long term benefits become unexpected. They also suggested using of triamcinolone as a long lasting CS in future studies (22).

Manfredini *et al.*, tried six different treatment protocols for patient with osteoarthritis to observe the improvement of nociceptive and functional symptoms (23). The drugs associated with AC were CS (triamcinolone) (group 2), low molecular weight HA (groups 3, 5, and 6), and high molecular weight HA (group 4). The treatments for group 4 resulted in unpleasant side effects which convinced authors to interrupt the treatment course. Because of the low intra articular space, the high molecular weight HA could not diffuse easily within the TMJ intra articular space. All other groups showed improvements in examined factors (MIO, chewing efficiency, pain at rest and in motion) with no significant differences (23).

In another study, Comert Kilic S studied the effect of AC with or without CS (methylprednisolone acetate) on osteoarthritis. The study was conducted on 32 joints of 24 patients and the final result revealed that the TMJ pain and joint sounds were statistically decreases in both groups, while the painless MIO was statistically improved only the CS group (13). The role of CS in improvement of MIO was similar between study groups in all of the reviewed articles except for Songul *et al.* who noted significant effect of AC with CS in MIO. That might be due to reduction of pain and inflammatory modulators which helps to release the adhered disk by elimination of negative pressure and surface friction (38).

Olsen-Bergem H and Bjørnland T (17) tried Vitamin B12 for AC in patients with Juvenile idiopathic arthritis. The Vitamin B12 is not generally considered as an anti-inflammatory agent. They did not specify the reason of using vitamin B12 during AC but they claimed that this treatment protocol is according to Alstergren P *et al.* study (39). During a meticulous overview on the Alstergren P *et al.* study, it was found that the Vitamin B12 was used as a mediator and indicator in measuring the interleukin 1β in the synovial fluid obtained from TMJs with arthritis (39). Olsen-Bergem H and Bjørnland T did not analyze any kind of inflammatory mediators in their methodology. Although the vitamin B12 might not have negative effects on the AC procedure or treatment sequence of TMDs, the researches supposed to be well aware about the purpose and properties of each drugs which is going to be administered in their study groups.

 Relying on the gathered information, most of the reviewed studies stated no significant effect of AC with CSs. The reason is not definite but some factors might be involved: the AC procedure before CSs injection might council the positive effects of CS (13), also the main reason of positive outcomes might be due to the pressure of lavage in AC not the pharmaceutical properties of used drugs (24). Moreover, direct comparison among studies in which AC has been administered for one of the study groups is not accurate enough because the AC technique is not standardized and inclusion criteria might differ from one study to another (20).

As mentioned before, the TMDs might have negative impacts on patient’s QoL. Tjakkes *et al.* overviewed the role of TMDs in patients’ QoL. They observed 95 patients and the results manifested that TMD pain less than one year did not influence the Qol significantly. However, in longer duration of pain, social function of patients was significantly decreased. In these cases, the mental health scores and emotional problems were not seriously affected (3). One of the reviewed studies, by Bouloux *et al.* (21), focused on the role of AC in patients’ QoL by evaluating some factors such as: Jaw Function Limitation Scale, MIO, mental health, and social functions. They revealed that the HA group showed an improvement in the physical health. Also, the control group (Ringer Lactate) showed an improvement in the mental health, however their results were not statistically significant. MIO without pain was improved among all of the studied groups with no significant differences. However, after one month follow up, the improvements were significant only in the CS and HA groups. The MIO results were similar after 3 months among all of the groups.

The side effects of AC:

The AC procedure is one of the minimally invasive surgical procedure. Nevertheless, The AC procedure might cause some side effects. Some of the reported complications are: pharyngeal edema, vascular and otologic and neurologic injuries, perforation of the middle cranial fossa, extradural hematoma (40). Ahmed *et al.* reported that one patient, out of 244 patients, was followed up for 24 h after TMJ AC due to vocal fold edema. They stated that through the medial joint capsule perforation following the extravasation of fluid was the main reason of edema (41). Nerve injuries mostly involve facial nerve injury (0.7–0.6 %), trigeminal nerve injury (0.1–2.4 %), otic injury (0.5–8.6 %) (42). Also, transient facial paralysis as a consequence of the local anesthetic infusion and the following alteration of motor function at the side of AC is one of the possible side effects (43).

Limitations:

One of the main obstacles upon us was the heterogeneous gathered data which prevented us to prepare a meta-analysis, however the results of reviewed studies were not too different form each other and making final conclusion was not difficult. The studies in which compare the other lavage agents such as HA were not available enough, either.

## Conclusions

According to the available RCTs on the role of CS during AC of TMJ, no significant result was found among CS groups and other groups (either control or other drugs). Although, reliable documents on effectiveness of CSs during AC of TMJ are not vast enough for making more determinant conclusion, it seems that CSs do not present better properties than other therapeutic drugs during AC. Nevertheless, dedicating more RCTs on this subject alongside with other methods and drugs are suggested in future researches.
